# Differential response of BDCA-1^+^ and BDCA-3^+^ myeloid dendritic cells to respiratory syncytial virus infection

**DOI:** 10.1186/1465-9921-14-71

**Published:** 2013-07-05

**Authors:** Meera R Gupta, Deepthi Kolli, Roberto P Garofalo

**Affiliations:** 1 Department of Internal Medicine, University of Texas Medical Branch, 301 University Blvd #0372, Galveston 77555, Texas; 2 Department of Pediatrics, University of Texas Medical Branch, 301 University Blvd #0372, Galveston 77555, Texas; 3 Department of Microbiology and Immunology, University of Texas Medical Branch, 301 University Blvd, Galveston 77555, Texas; 4 Sealy Center for Molecular Medicine, University of Texas Medical Branch, Galveston 77555, Texas

**Keywords:** RSV, Myeloid dendritic cells, BDCA-1^+^ mDCs, BDCA-3^+^ mDCs, Primary DCs, Immune response, Costimulatory molecules, CD80, CD86, PD-L1, Cytokine profiles

## Abstract

**Background:**

Respiratory syncytial virus (RSV) is the leading cause of respiratory infections in children, elderly, and immunocompromised individuals. Severe infection is associated with short- and long-term morbidity including pneumonia, recurrent wheezing, and abnormal pulmonary function, and several lines of evidence indicate that impaired adaptive immune responses during infection are critical in the pathophysiology of RSV-mediated disease. Myeloid Dendritic cells (mDCs) play a pivotal role in shaping antiviral immune responses in the respiratory tract; however, few studies have examined the interactions between RSV and individual mDC subsets. In this study, we examined the effect of RSV on the functional response of primary mDC subsets (BDCA-1^+^ and BDCA-3^+^) isolated from peripheral blood.

**Methods:**

BDCA-1^+^ and BDCA-3^+^ mDCs were isolated from the peripheral blood of healthy adults using FACS sorting. Donor-matched BDCA-1^+^ and BDCA-3^+^ mDCs were infected with RSV at a multiplicity of infection (MOI) of 5 for 40 hours. After infection, cells were analyzed for the expression of costimulatory molecules (CD86, CD80, and PD-L1), cytokine production, and the ability to stimulate allogenic CD4^+^ T cell proliferation.

**Results:**

Both BDCA-1^+^ and BDCA-3^+^ mDCs were susceptible to infection with RSV and demonstrated enhanced expression of CD86, and the inhibitory costimulatory molecules CD80 and PD-L1. Compared to BDCA-3^+^ mDCs, RSV-infected BDCA-1^+^ mDC produced a profile of cytokines and chemokines predominantly associated with pro-inflammatory responses (IL-1β, IL-6, IL-12, MIP-1α, and TNF-α), and both BDCA-1^+^ and BDCA-3^+^ mDCs were found to produce IL-10. Compared to uninfected mDCs, RSV-infected BDCA-1^+^ and BDCA-3^+^ mDCs demonstrated a reduced capacity to stimulate T cell proliferation.

**Conclusions:**

RSV infection induces a distinct pattern of costimulatory molecule expression and cytokine production by BDCA-1^+^ and BDCA-3^+^ mDCs, and impairs their ability to stimulate T cell proliferation.

The differential expression of CD86 and pro-inflammatory cytokines by highly purified mDC subsets in response to RSV provides further evidence that BDCA-1^+^ and BDCA-3^+^ mDCs have distinct roles in coordinating the host immune response during RSV infection. Findings of differential expression of PD-L1 and IL-10 by infected mDCs, suggests possible mechanisms by which RSV is able to impair adaptive immune responses.

## Introduction

Respiratory Syncytial Virus (RSV), a ssRNA pneumovirus of the *paramyxoviridae* family, is the leading cause of acute lower respiratory tract infections in children, elderly, and immunocompromised patients. More than 95% of children become infected with RSV by 2 years of age, and approximately 50% of children infected in the first year of life are reinfected during the second year [1]. Reinfection continues throughout life, re-emerging as a major cause of community-acquired pneumonia in the elderly [2]. RSV infection is associated with short- and long-term morbidity including pneumonia, recurrent wheezing, and abnormal pulmonary function. Moreover, RSV bronchiolitis has been identified as the highest independent risk factor for the development of asthma and allergic sensitization later in life [3].

Primary RSV infection is controlled by T cell responses which are crucial in viral clearance and limiting disease severity. Weak antiviral cytotoxic responses and decreased numbers of CD4^+^ and CD8^+^ T cells in the lower respiratory tract of infants with severe infection [4,5] along with the susceptibility to repeat infections with RSV strongly suggest that the failure to develop adaptive T cell responses is critical in the pathophysiology of RSV-mediated disease. Additionally, the balance between Th1 and Th2 cytokines produced by T cells plays a crucial role in determining the outcome of infection, and severe RSV infection may be associated with skewing the Th1/Th2 balance of the virus-specific response towards Th2 [6,7]. However, the mechanisms by which RSV is able to manipulate host immunity are not fully understood.

Dendritic cells (DCs) are an important first line of defense against invading pathogens and are regarded as the most potent antigen presenting cell (APC) type. Upon antigen recognition, DCs produce cytokines and costimulatory signals needed to guide T cell differentiation, ultimately determining the quality and quantity of the resulting immune response. Human DC populations are defined based on their lineage and expression of unique Blood Dendritic Cell Antigens (BDCA), and depending on their derivation, direct immune responses through either the initiation or modulation of immunity. Plasmacytoid DCs (pDCs), characterized by expression of BDCA-2, mediate antiviral immunity via the production of IFN-α [8]. Myeloid DCs (mDCs) are particularly efficient in the uptake, processing and presentation of antigens and can be further subdivided into distinct subsets identified by differential expression of either BDCA-1 (BDCA-1^+^) or BDCA-3 (BDCA-3^+^).

The lung contains an elaborate network of dendritic cells that regulate the local environment. During respiratory infections, both tissue-resident DCs, as well as recruited DCs, are activated as part of the host immune response [9]. Although present at much lower frequencies than BDCA-1^+^ mDCs in peripheral blood, the DC network in the airway mucosa is comprised predominately of BDCA-3^+^ mDCs [10]. Studies of mDC subsets isolated from human lungs and blood suggest that each subset exhibits distinct roles in coordinating the immune response against pathogens [11-14]. Although the relationship between mDCs found in the lung and peripheral blood is not clear, parallel phenotypic analysis and transcriptome mapping provides evidence that lung and other non-lymphoid tissue BDCA-3^+^ mDCs are potentially related to blood BDCA-3^+^ mDCs [15]. The relationship of blood to tissue BDCA-1^+^ mDCs was not as clearly defined, but did show clustering of their gene signatures [15]. Thus, even though it is likely that lung DCs may in some ways be functionally distinct from blood DCs, these studies demonstrate that blood mDC subsets are potentially related to mDC subsets found in tissues.

As key regulators of immunity, DCs are an ideal target for the virus to exert its immune altering mechanisms. In this regard, a possible role for mDCs in RSV infection is evidenced by observations of increased numbers of mDCs in the airways, and decreased numbers of mDCs in the blood of children with acute RSV infection, suggesting that DCs are recruited out of the blood to the site of infection [16]. Correlations between the number of mDCs and the level of pro-inflammatory cytokines in the nasal cavity of RSV-infected patients indicates that mDCs may represent an important source of inflammatory cytokines during the host immune response against infection [16]. Additionally, RSV infection inhibits the capacity of monocyte derived DCs (Mo-DCs) to stimulate T-cell proliferation *in vitro*[17,18] and impairs the function of pulmonary DCs in RSV-infected mice [19]. Taken together, these results suggest that infection with RSV impairs the ability of host immune response to adequately clear viral pathogens by altering DC mediated immune responses.

However, current RSV-mDC studies often utilize murine DCs or mDCs derived *in vitro* from human monocytes, and even though Mo-DCs have many characteristics similar to myeloid blood DCs [20-22], studies have not shown direct functional correlations between *in vitro* derived Mo-DCs and individual mDC subsets isolated from lymph nodes or blood [14,23,24]. Thus, studies using Mo-DCs may not adequately recapitulate the function of primary mDCs during RSV infection. Few studies have examined the interactions between viral pathogens and primary human mDC subsets, especially BDCA-3^+^ mDCs. To identify cell-specific responses of primary mDC subsets to RSV infection, we examined the expression of costimulatory markers and production of cytokines by BDCA-1^+^ and BDCA-3^+^ mDCs after exposure to RSV. The functional response after infection was evaluated by examining their ability to stimulate T cell proliferation in a mixed lymphocyte reaction. To our knowledge, this is one of the first studies to examine the functional response of highly purified BDCA-1^+^ and BDCA-3^+^ mDCs to RSV.

## Materials and methods

### Isolation of mDC subsets

Buffy coats prepared by the University of Texas Medical Branch (UTMB) blood blank from healthy adult donors who fulfilled criteria for blood donations were obtained. The donor’s identity, race, and age remained anonymous to investigators. This study was approved by UTMB’s Institutional Review Board. Peripheral blood mononuclear cells (PBMCs) were separated from buffy coats by Ficoll-hypaque density centrifugation and enriched for mDCs using an mDC cell isolation kit (Miletnyi Biotec, Auburn, CA) to deplete magnetically labeled non-mDCs. In preparation for FACS sorting, the DC enriched fraction was stained with the following fluorochrome-conjugated monoclonal antibodies (mAbs[clone]): PE-BDCA-3[1A4], APC-BDCA-1[AD5-8E7], FITC-BDCA-2[AC144], and FITC-lineage marker cocktail containing CD3[SK7], CD14[MΦP9], CD16[3G8], CD19[SJ25C1], CD20[L27], CD56[NCAM16.2] purchased from eBiosciences (San Diego, CA), Miltenyi Biotec, and BD Biosciences (San Jose, CA) respectively. Cells were stained in the presence of FcR-blocking reagent (Miltenyi Biotec) to minimize non-specific binding.

Sorting and flow cytometric data acquisition was performed on a FACSAria (BD Biosciences) capable of 9-color analysis running FACS Diva software. BDCA-3^+^ mDCs were sorted as FITC^-^, APC^-^, PE^hi^ and BDCA-1^+^ were sorted as FITC^-^, APC^+^, PE^dim^ with routine post-sort analysis to ensure purity ≥95%.

### Virus preparation and infection

Preparation of sucrose-purified RSV (Long strain) has been previously described [18]. RSV was inactivated by exposing the virus to ultraviolet light [18]. For infection, 1×10^4^ cells of donor-matched BDCA-1^+^ or BDCA-3^+^ mDCs were resuspended in 200 μl of cRPMI (RPMI 1640 medium + 2 mmol/liter L-glutamine + 10% FBS + 50 μM 2-ME + 100UI/ml penicillin-streptomycin) and incubated for 40 hours at 37°C with RSV or ultraviolet-inactivated RSV (UV-RSV) at a multiplicity of infection (MOI) of 5 [18,25]. As TLR3 is known to be expressed on BDCA-1^+^ and BDCA-3^+^ mDCs [13], cells cultured in the presence of 10 μg/ml of purified poly I:C (InvivoGen, San Diego, CA) served as positive controls and uninfected cells served as negative controls.

### FACS analysis of DCs

Mock and RSV-infected BDCA-1^+^ and BDCA-3^+^ mDCs were first stained with Fixable Viability Dye eFlour 780 (eBioscience). To detect intracellular expression of RSV antigens, BDCA-1^+^ and BDCA-3^+^ mDCs were fixed with Cytofix/Cytoperm™ (Pharmingen), permeabilized with Perm/Wash™ buffer (Pharmingen), and stained with FITC-conjugated anti-RSV antibody (Biosource, Camarillo, CA) as previously described [18]. For costimulatory molecule analysis, mDCs were stained with FITC-PD-L1[M1H1], v450-CD86[2331(FUN-1)], and PE-Cy7-CD80[L307.4] (BD Biosciences) or in a separate set of experiments, PerCP Cy5.5-HLA-DR[L243]. Flow cytometric data acquisition was performed on a BD LSRII Fortessa capable of 18-color analysis running FACS Diva software (BD Biosciences). FloJo V7.6.3 software was used to analyze all flow cytometry data.

### Bio-plex assay

After 40 hours, cell free supernatants were collected and tested for cytokines IL-1β, IL-1rα IL-6, IL-7, IL-8, IL-10, IL-12(p70), IFN-γ, CXCL10, TNF-α, G-CSF, MCP-1, MIP-1α, MIP-1β, and RANTES using the Luminex-based Bio-Plex system (Bio-Rad Laboratories, Hercules, CA). The lower limit of detection for all cytokines measured in this assay is 3 pg/ml. When comparing cytokine production between subsets, the fold change in concentration (infected/uninfected) was used to normalize results across cell types.

### Allogenic MLR

Allogenic CD4^+^ T cells were isolated from PBMCs by negative immunomagnetic selection using a CD4^+^ T cell isolation kit (Miletnyi Biotec, Auburn, CA). Routine post sort purity was >96% as determined by flow cytometry. To track proliferation, T cells were labeled with 10 μM CFSE (Invitrogen, Grand Island, NY) according to the manufacturer’s instructions. Donor matched BDCA-1^+^ and BDCA-3^+^ mDCs were incubated for 24 hours at 37°C with RSV, UV- RSV, or poly I:C. After 24 hours, cells were washed and cocultured with the CFSE-labeled allogenic T cells at a ratio of 1:5 in RPMI containing 5% FCS for 6 days at 37°C. T cells alone and T cells incubated with soluble CD3 and CD28 were included as controls. Prior to flow cytometry, cells were stained with PerCP-Cy™ 5.5-CD3[SK7] and PE-Cy™ 7-CD4[RPT-A] (BD Biosciences). Proliferation of CD3^+^ CD4^+^ T cells was measured on a BD LSRII Fortessa running FACS Diva software (BD Biosciences) and analyzed using FloJo V7.6.3 software.

### Statistical analysis

Statistical Analysis was performed with InStat 5.02 biostatistics package (GraphPad Software, San Diego, CA) using student’s paired t-tests to ascertain differences between uninfected and infected cells of the same type or student’s t-tests to ascertain differences between cell types. Significance was defined as p ≤0.05. Prior to analysis, data sets were log transformed to normalize non-normally distributed data.

## Results

### Isolation and characterization of mDC subsets

**Figure 1:**
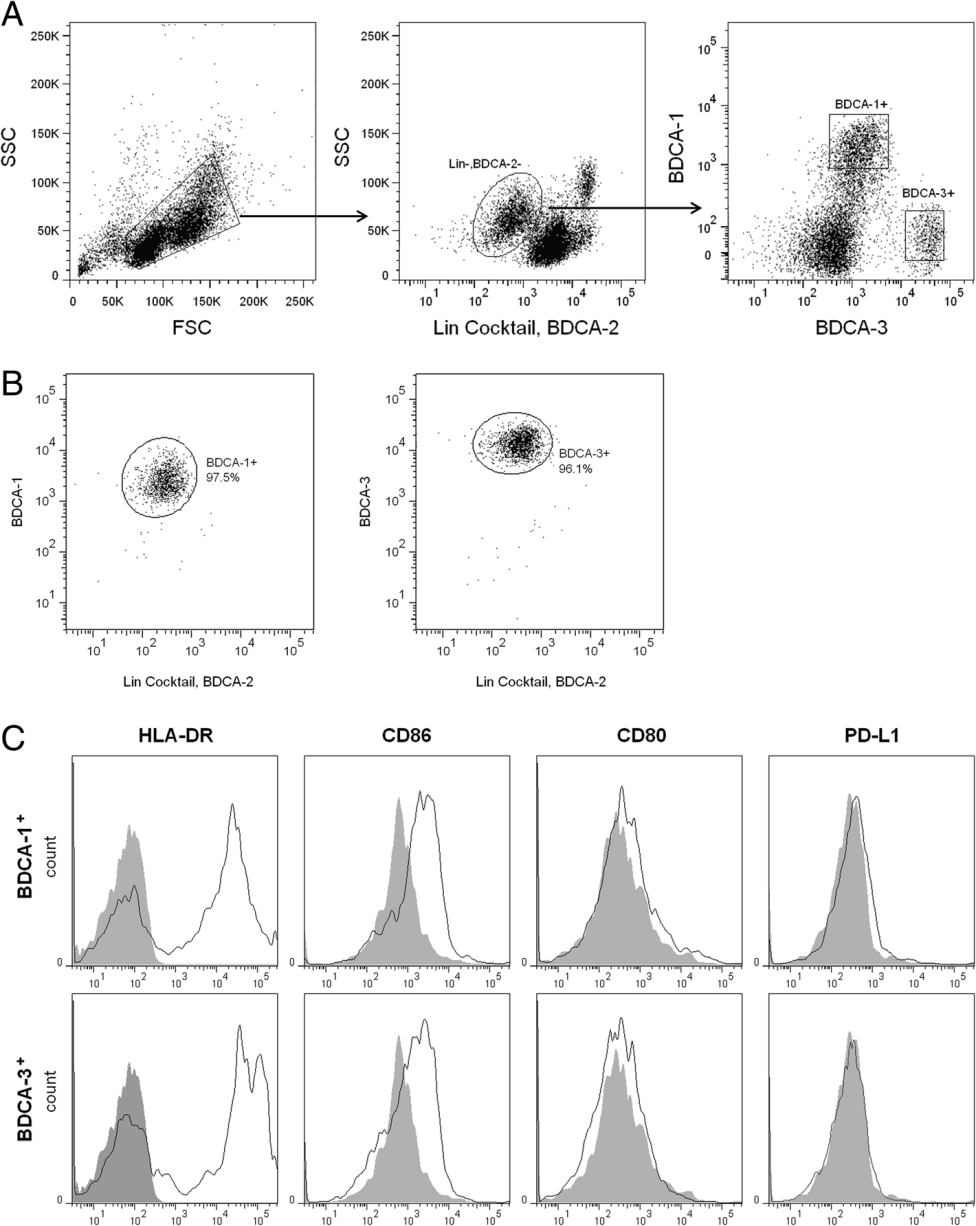
**Isolation and phenotypic characterization of mDC subsets. (A)** PBMCs were gated on SSC and FSC to exclude dead cells and debris. Cells were then gated to exclude lineage positive cells and pDCs. Lineage (Lin)^-^, BDCA-2^-^ cells were then gated for expression of BDCA-1 and BDCA-3. BDCA-1^+^ mDCs were sorted as Lin^-^, BDCA-2^-^, BDCA-1^+^, BDCA-3^dim^ and BDCA-3^+^ mDCs were sorted as Lin^-^, BDCA-2^-^, BDCA-1^-^, BDCA-3^hi^. **(B)** Post-sort purity for all sorted cells was ≥95%. Data is from one donor and is representative of 6 donors. **(C)** Expression of maturation and costimulatory molecules by freshly isolated cells. Filled curves= isotype, solid line=mDC. Data is from one donor and is representative of 3 donors.

MDC subsets were isolated from PBMCs using a two step procedure [13]. PBMCS were first enriched for mDCs using a myeloid dendritic cell isolation kit to deplete non-myeloid DCs via negative immunoselection, followed by flow cytometry to eliminate residual pDCs and lineage positive cells and sort for BDCA-1^+^ and BDCA-3^+^ cells (Figure 1A). Consistent with reported frequencies in blood [13], we were routinely able to isolate approximately 1×10^4^ BDCA-3^+^ mDCs per 1×10^8^ PBMCS and 1×10^5^ BDCA-1^+^ mDCs per 1×10^8^ PBMCs with a post-sort purity ≥95% (Figure 1B). At baseline, both subsets demonstrated similar expression of HLA-DR and CD86, but did not express CD80 or PD-L1 (Figure 1C).

### RSV infects BDCA-1^+^ and BDCA-3^+^ mDCs

**Figure 2:**
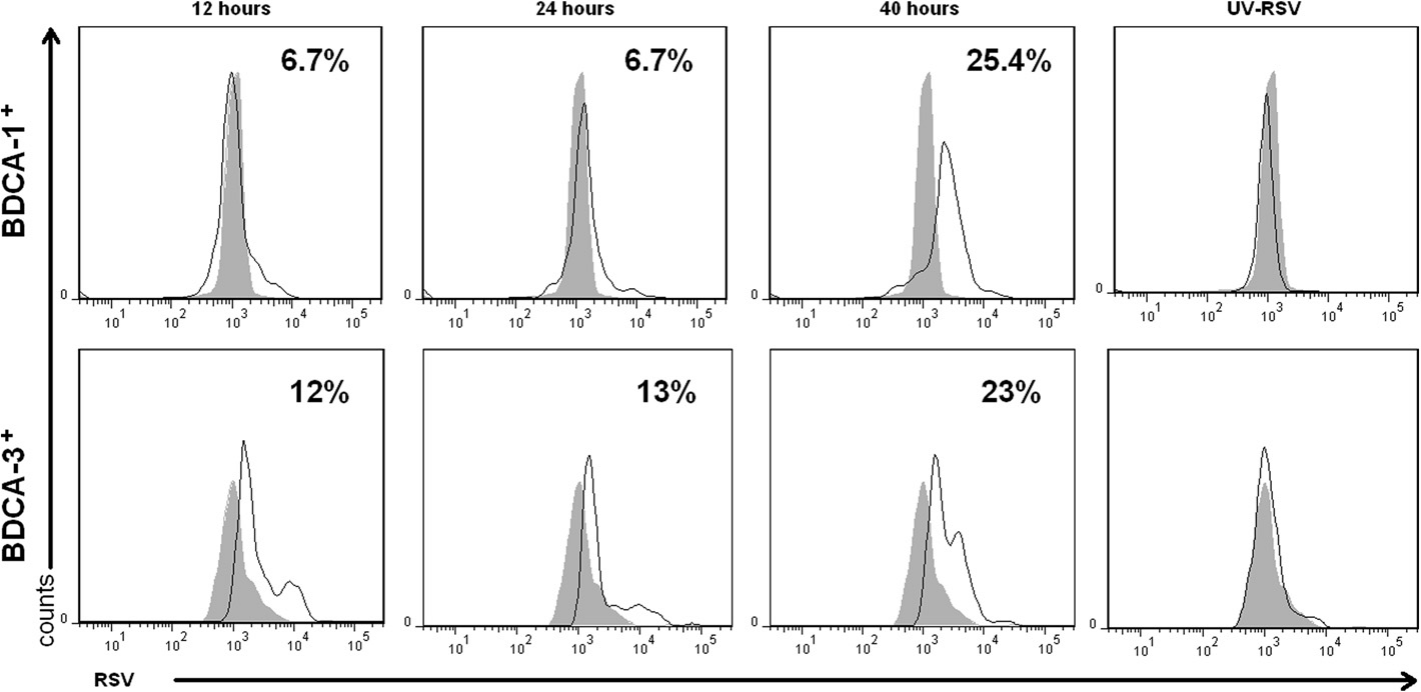
**BDCA-1**^**+ **^**and BDCA-3**^**+ **^**mDCs are susceptible to infection with RSV.** BDCA-1^+^ and BDCA-3^+^ mDCs were incubated in media alone or exposed to RSV and UV-inactivated RSV at a MOI=5 for 12, 24, or 40 hours. Cells were stained for intracellular expression of RSV protein, and the percentage of RSV-infected cells was quantified using flow cytometry to detect expression of anti-RSV antibody. Filled grey curve=uninfected cells, solid line=infected cells. Data is from one donor, and is representative of 3 donors.

Work from our lab has previously shown that human Mo-DCs are permissive to RSV infection [18]. To determine the susceptibility of primary mDCs to infection, the intracellular expression of viral proteins by BDCA-1^+^ and BDCA-3^+^ infected with RSV at a MOI=5 for 12, 24, or 40 hours was examined using flow cytometry. The greatest percentage of infected cells was detected at 40 hours, with 20-25% of BDCA-1^+^ mDCs and 18-23% of BDCA-3^+^ mDCs demonstrating expression of viral proteins (Figure 2). Cells exposed to UV-RSV (non-replicating virus) did not express RSV antigen. Cell viability decreased between 24 and 40 hours; however, there was no difference in the number of viable cells after exposure to RSV and UV-RSV at each time point. In BDCA1^+^ mDCs, maximal infection occurred at a MOI=5 and increasing the MOI did not result in a further increase in the number of virus-positive cells, whereas, in BDCA-3^+^ mDCs using an MOI=10 did increase the number of virus-positive cells by approximately 4% (data not shown). As there was no statistically significant difference between the rates of infection in BDCA-1^+^ and BDCA-3^+^ mDCs at a MOI=5, this MOI was used for all subsequent RSV infection experiments.

### RSV infection modulates costimulatory molecule expression

**Figure 3:**
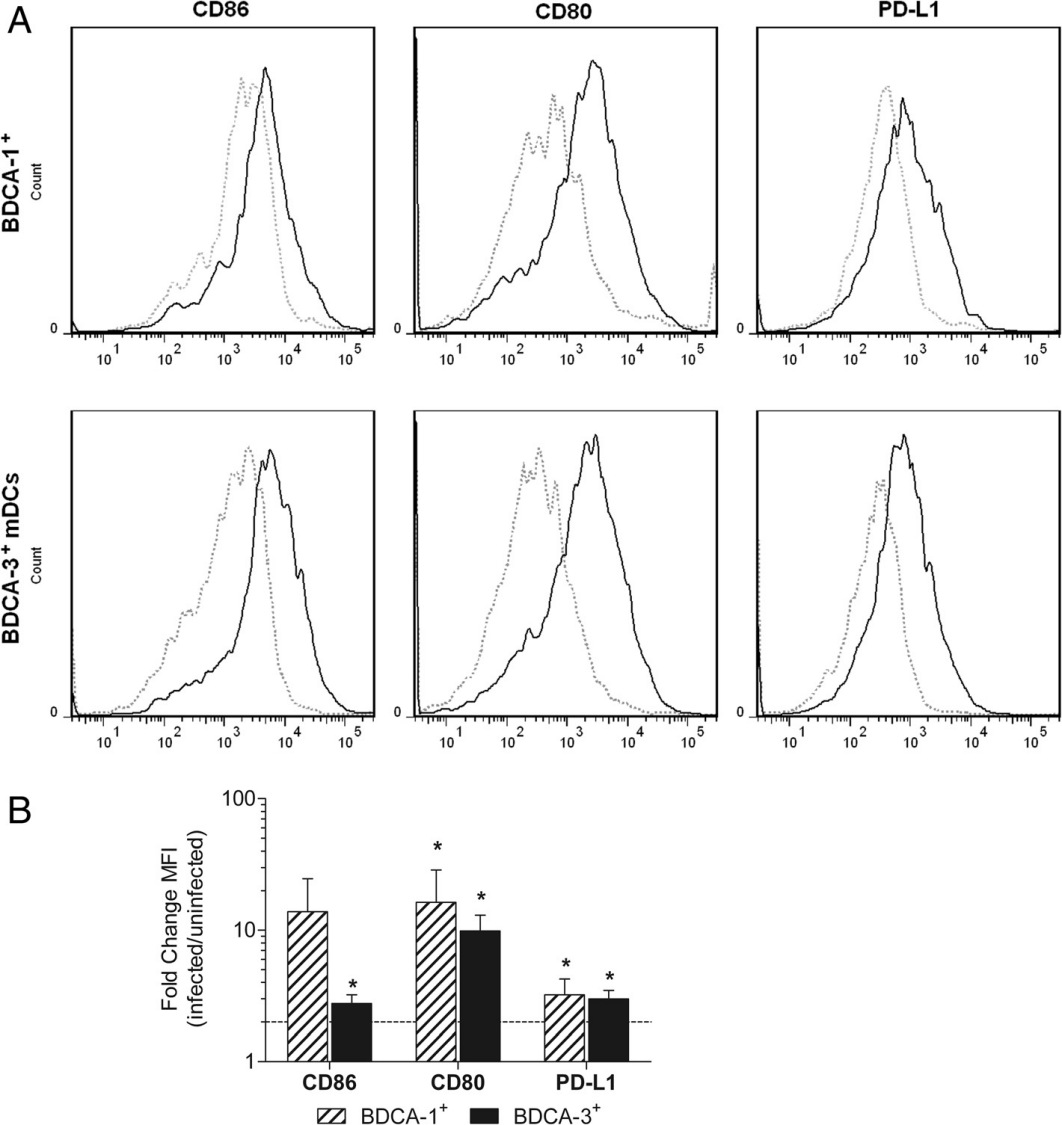
**RSV infection induces upregulation of costimulatory molecules on BDCA-1**^**+ **^**and BDCA-3**^**+ **^**mDCs.** BDCA-1^+^ and BDCA-3^+^ mDCs were incubated in media alone or infected with RSV at a MOI=5 for 40 h. **(A)** Costimulatory molecule expression was analyzed using flow cytometry. Dashed line= uninfected cells, solid line = infected cells. Data is from one donor and is representative of 5 donors. **(B)** Bar graph represent the change in mean fluorescent intensity (MFI) of expression by infected cells over uninfected cells (mean + SEM of 5 donors). Linear data is reported on a logarithmic scale. The dotted line represents a 2 fold induction. *=statistically significant difference (*p≤0.05*) in expression by infected mDCs as compared to uninfected mDCs by student’s paired *t*-test.

T cell activation depends, in addition to the binding of MHC/peptide complexes by T cell receptors, on the recognition of positive or negative costimulatory molecules expressed by DCs. Depending on whether they bind to CD28 or CTLA-4 receptors on T cells, CD86 and CD80 expressed on mDCs can deliver either stimulatory (CD28) or inhibitory (CTLA-4) signals for T cell activation. Additionally, the programmed death-1 (PD-1) receptor and its ligands PD-L1 and PD-L2 deliver inhibitory signals that regulate the balance between tolerance vs. T cell activation and immune-mediated tissue damage. Engagement of PD-1 by its ligands blocks T cell proliferation and cytokine production [26] and promotes the development, expansion, and survival of Tregs [27,28]. Previous work from our lab demonstrated increased expression of CD86, CD80, and PD-L1, but not PD-L2, on RSV-infected Mo-DCs [18,19]. Therefore, to assess the effect of RSV infection on the regulation of costimulatory molecule expression in primary mDC subsets, the expression of CD86, CD80, and PD-L1 was examined on RSV infected BDCA-1^+^ and BDCA-3^+^ mDCs using flow cytometry. In all donors, both RSV-infected BDCA-1^+^ and BDCA-3^+^ mDCs demonstrated enhanced expression of CD86, CD80, and PD-L1 as compared to uninfected cells (Figure 3A). However, CD86 expression by RSV-infected BDCA-1^+^ mDCs was not found to be significantly different from expression by uninfected cells (Figure 3B). Exposure to UV-RSV also induced expression of CD86 and CD80 in BDCA-1^+^ and BDCA-3^+^ mDCs, but did not induce expression of PD-L1 in either subtype (Additional file 1: Figure S1).

**Figure 4:**
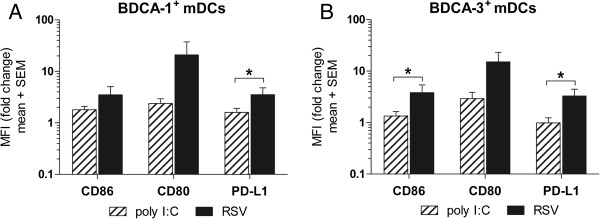
**Comparison of costimulatory molecule expressed by mDC subsets in response to RSV and poly I:C. (A)** BDCA-1^+^ and **(B)** BDCA-3^+^ mDCs were incubated in media alone, infected with RSV at a MOI=5, or activated with poly I:C for 40 h. Bar graphs represent the change in mean fluorescent intensity (MFI) of expression by infected cells over uninfected cells (mean + SEM of 5 donors). Linear data is reported on a logarithmic scale. The dotted line represents a 2 fold induction. *=statistically significant difference (*p≤0.05*) in expression by RSV-infected mDCs as compared to poly I:C-activated mDCs by student’s paired *t*-test.

DC subsets express discreet TLR profiles [13,29], however both BDCA-1^+^ and BDCA-3^+^ mDCs have been shown to express TLR3 [13]. Activation with poly I:C upregulates the expression of costimulatory molecules on BDCA-1^+^ and BDCA-3^+^ mDCs (Additional file 2: Figure S2A-B). To characterize the strength and specificity of costimulatory molecule expression induced by RSV, the fold change in CD86, CD80, and PD-L1 expression by mDCs infected with RSV was compared to mDCs activated with poly I:C. In BDCA-1^+^ mDCs, PD-L1 expression was significantly greater on RSV-infected cells than poly I:C activated cells (Figure 4A); in BDCA-3^+^ mDCs, expression of CD86 and PD-L1 was significantly greater on RSV-infected cells than poly I:C activated cells (Figure 4B). Together, these results suggest although viral replication is not necessary for mDC activation; PD-L1 is differentially induced by RSV infection.

### RSV infection induces cytokine production by BDCA-1^+^ and BDCA-3^+^ mDCs

**Figure 5:**
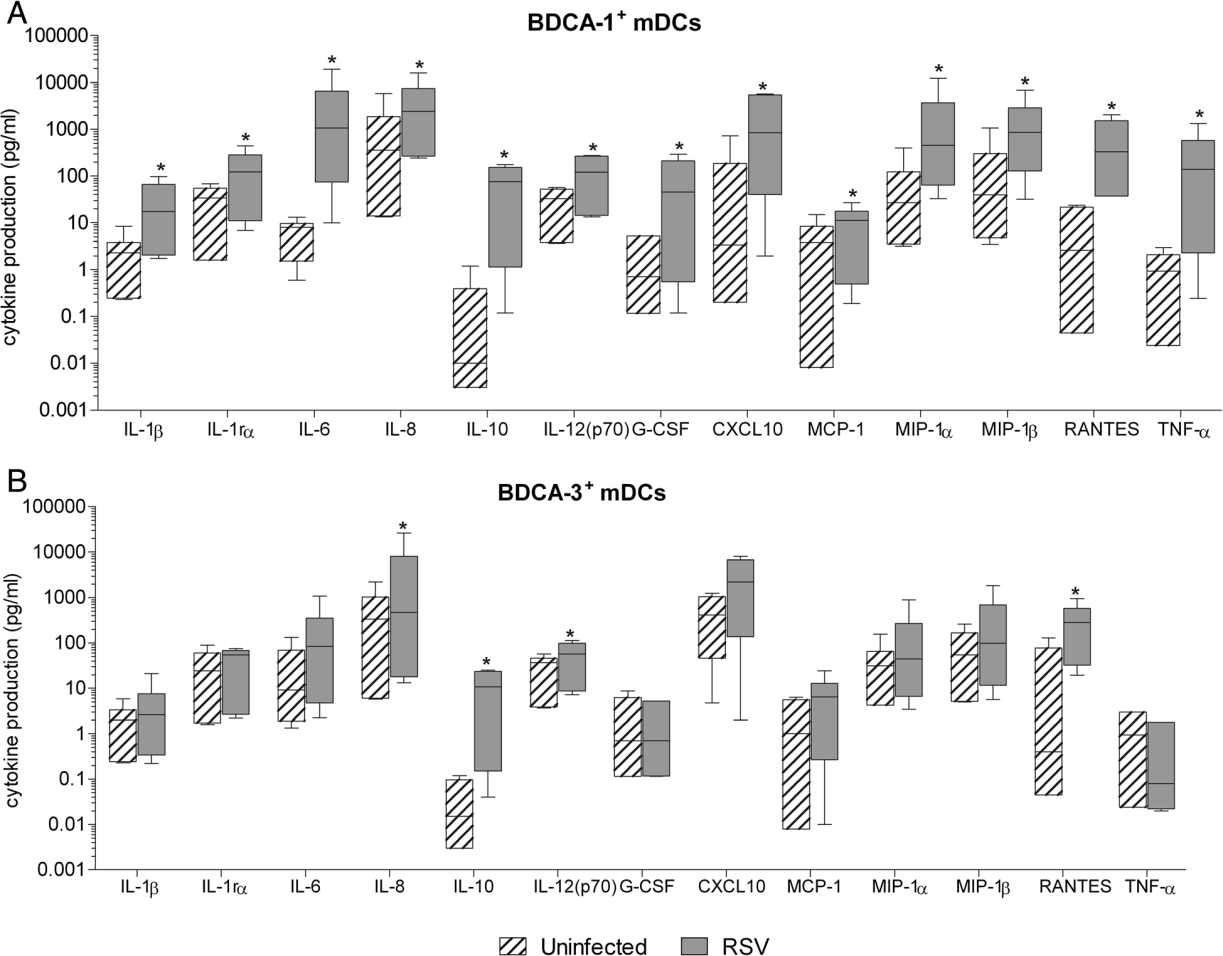
**RSV infection induces cytokine production by BDCA-1**^**+ **^**and BDCA-3**^**+ **^**mDCs.(A)** BDCA-1^+^ and **(B)** BDCA-3^+^ mDCs were isolated and incubated with RSV or media for 40 hours. Cytokine and chemokine levels were measured in cell-free supernatant by multiplex assay. Data represent cytokine production in pg/ml. Box and whisker plots show the median (central bar), interquartiles (boxes), and range (whiskers) of the data from 6 donors. Linear data is reported on a logarithmic scale. *= Statistically significant *(p≤0.05)* difference in cytokine concentration between infected and uninfected cells as calculated by student’s paired *t*-test.

The cytokines and chemokines produced by activated mDCs affect both innate and adaptive immune responses. To examine the profile of cytokines produced by primary mDC subsets in response to RSV, cytokine production by RSV-infected BDCA-1^+^ and BDCA-3^+^ mDCs was examined using a Luminex-based assay. Compared to uninfected cells of the same type, RSV-infected BDCA-1^+^ mDCs demonstrated significant increases in the production of IL-1β, IL-1rα, IL-6, IL-8, IL-10, IL-12, G-CSF, TNF-α, CXCL10, MCP-1, MIP-1α, MIP-1β, and RANTES (Figure 5A). In three of the six donors, IFN-γ was also produced by RSV-infected BDCA-1^+^ mDCs, with high levels produced by only one of the three (data not shown). The significance of these findings is unclear since with the exception of IFN-γ, the pattern of production for all other cytokines was consistent among most donors. In RSV-infected BDCA-3^+^ mDCs, IL-8, IL-10, IL-12, and RANTES levels were found to be significantly increased as compared to uninfected cells of the same type (Figure 5B), however for most donors, the amount of IL-12 produced by RSV-infected BDCA-3^+^ mDCs did not reach a 2 fold increase from uninfected cells. IFN-γ was not produced by RSV-infected BDCA-3^+^ mDCs in any of the six donors. Overall, RSV induced a more robust cytokine response from BDCA-1^+^ mDCs compared to BDCA-3^+^ mDCs. With the exception of IL-12 and IP-10, exposure to both RSV and UV-RSV induced cytokine production in BDCA-1^+^ and BDCA-3^+^ mDCs (data not shown) suggesting that as in Mo-DCs, [30,31] viral replication is not necessary for mDC activation. IFN-α is a key mediator of anti-viral immunity. In a preliminary set of experiments with three donors, production of IFN-α by RSV-infected BDCA-1^+^ and BDCA-3^+^ mDCs was measured using ELISA. Neither BDCA-1^+^ or BDCA-3^+^ mDCs from any of the three donors produced detectable levels of IFN-α (data not shown). As these results coincided with previous findings in RSV-infected Mo-DCs [18], no further donors were tested.

**Figure 6:**
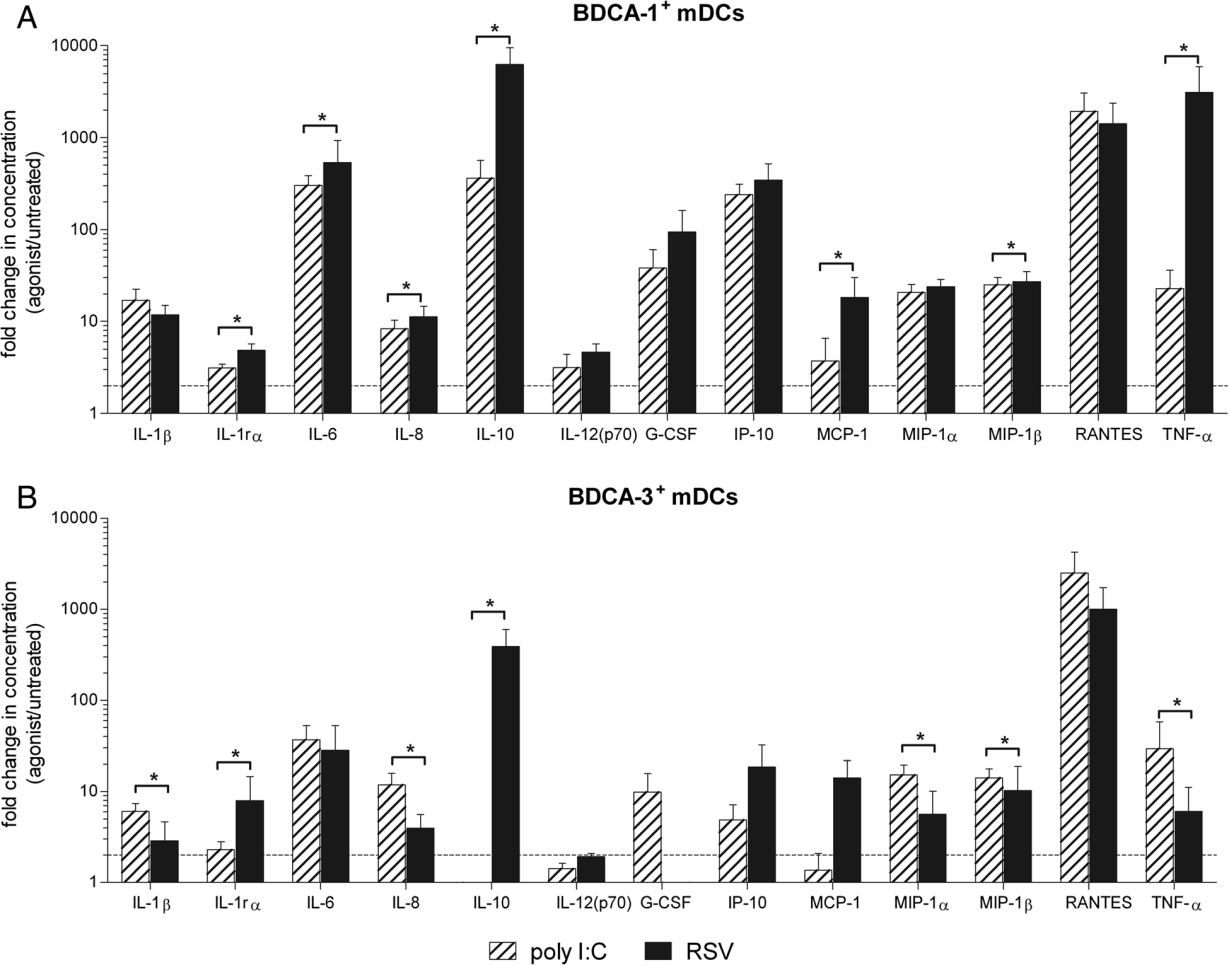
**Comparison of cytokines produced by mDC subsets in response to RSV and poly I:C. (A)** BDCA-1^+^ and **(B)** BDCA-3^+^ mDCs were isolated and incubated with RSV, poly I:C or media alone for 40 hours. Cytokine and chemokine levels were measured in cell-free supernatant by multiplex assay. Data represents the mean + SEM of the fold change in cytokine production by treated cells as compared to untreated cells of the same type from 5 donors. Linear data is reported on a logarithmic scale. The dotted line represents a 2 fold induction. *= Statistically significant *(p≤0.05)* difference in cytokine production by RSV-infected mDCs as compared to poly I:C-activated mDCs by student’s paired *t*-test.

BDCA-1^+^ and BDCA-3^+^ mDCs produce proinflammatory cytokines in response to activation with poly I:C (Additional file 3: Figures S3A and 3B). To characterize the strength and specificity of the cytokines produced by RSV-infected BDCA-1^+^ and BDCA-3^+^ mDCs, the fold change (agonist/untreated) in cytokine production in response to RSV and poly I:C was compared for each subset. Although the cytokine profiles produced by BDCA-1^+^ mDCs in response to RSV and poly I:C were similar, RSV-infected BDCA-1^+^ mDCs did produce significantly greater amounts of IL-1rα, IL-6, IL-8, IL-10, MCP-1, MIP-1β, and TNF-α (Figure 6A). As compared to activation with poly I:C, BDCA-3^+^ mDCs infected with RSV produced significantly greater amounts of IL-1rα and IL-10, but decreased amounts of IL-1β, IL-8, MIP-1α, MIP-1β, and TNF-α (Figure 6B).

**Figure 7:**
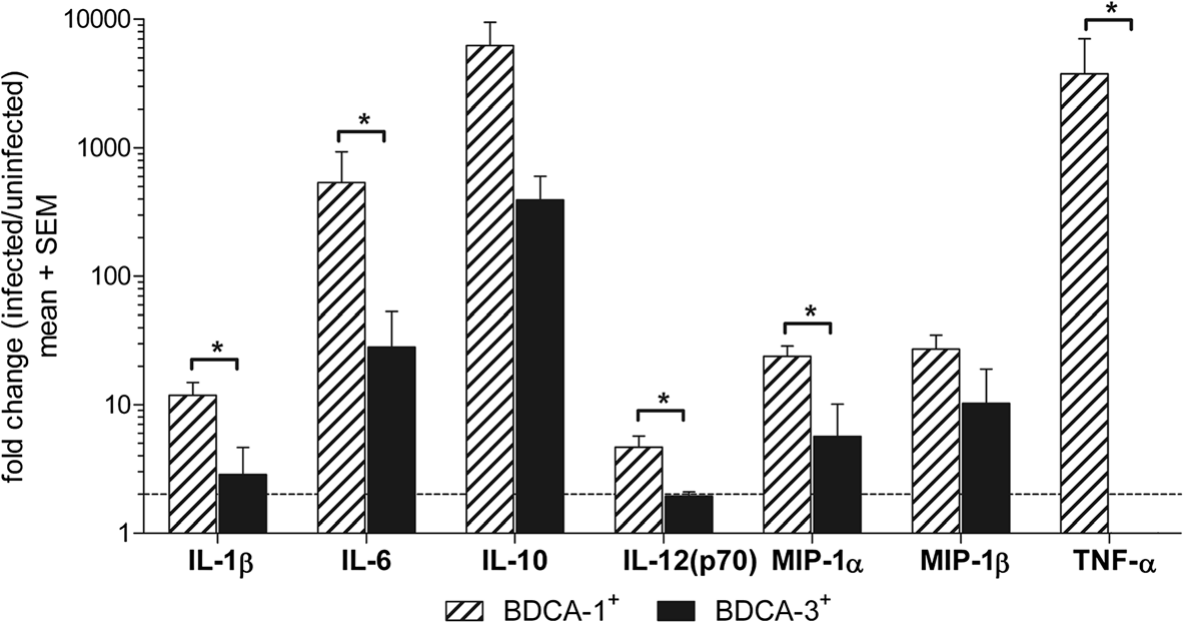
**Comparison of cytokines produced by RSV-infected BDCA-1**^**+ **^**and BDCA-3**^**+ **^**mDCs.** DC subsets were isolated and incubated with RSV or media for 40 hours. Cytokine and chemokine levels were measured in cell-free supernatant by multiplex assay. Data represents the mean + SEM of the fold change in cytokine production by infected cells as compared to uninfected cells of the same type from 6 donors. Linear data is reported on a logarithmic scale. The dotted line represents a 2 fold induction. *= Statistically significant *(p≤0.05)* difference in cytokine production between RSV-infected BDCA-1^+^ and BDCA-3^+^ mDC as calculated by students *t*-test.

IL-12 and IFN-γ promote Th1 polarization [32]. The cytokine signature associated with Th2 skewing is not as clearly defined; however along with IL-4, IL-10 and low levels of IL-12 have been identified as Th2 polarizing signals [32,33]. Other pro-inflammatory cytokines and chemokines also regulate T cell differentiation, and IL-1β, IL-6, TNF-α, MIP-1α, and MIP-1β are reported to have important roles in promoting DC maturation and inducing Th1 responses [34-36]. To determine whether the cytokines and chemokines associated with polarization of T cell responses were differentially induced by RSV-infected BDCA-3^+^ and BDCA-1^+^ mDCs, the fold change (infected/uninfected) in production of these cytokines by each cell type was compared (Figure 7). In general the fold changes in cytokine and chemokine production were lower in BDCA-3^+^ mDCs compared to BDCA-1^+^ mDCs, which may in part be related to the slightly higher levels of cytokine production by uninfected BDCA-3^+^ mDCs. Compared to BDCA-3^+^ mDCs, RSV-infected BDCA-1^+^ mDC were found to produce significantly increased amounts of IL-1β, IL-6, IL-12, MIP-1α, and TNF-α; production of MIP-1β and IL-10 was not significantly different between the two subsets. Neither BDCA-1^+^ or BDCA-3^+^ mDCs were found to produce IL-4.

### RSV infection impairs the capacity of BDCA-1^+^ and BDCA-3^+^ mDCs to stimulate T cell proliferation

**Figure 8:**
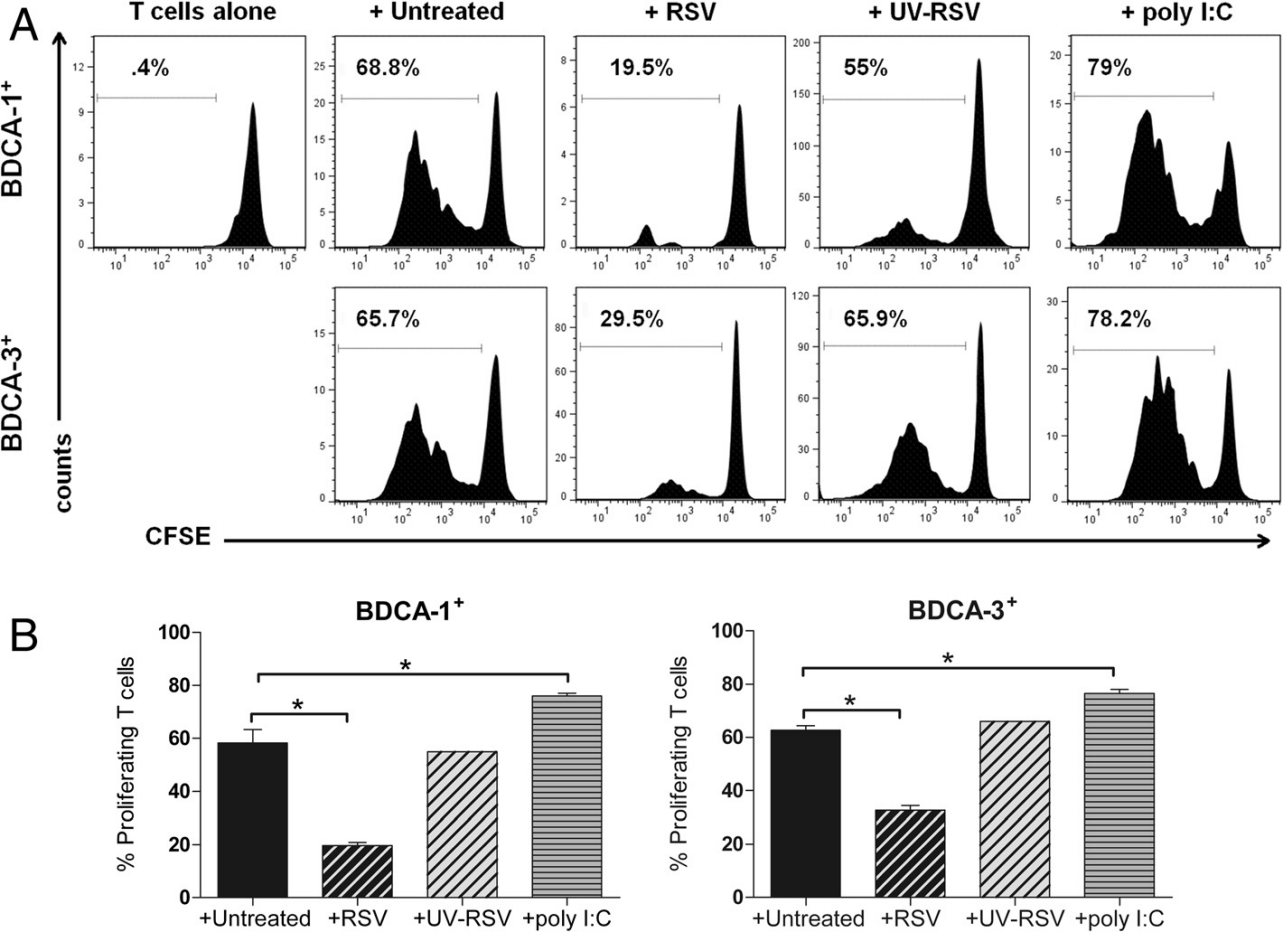
**RSV-infected BDCA-1**^**+ **^**and BDCA-3**^**+ **^**mDCs impair CD4**^**+ **^**T cell proliferation.** BDCA-1^+^ and BDCA-3^+^ mDC were incubated with RSV, UV-RSV, poly I:C or media for 24 hours. Cells were washed and cocultured with CFSE-labeled allogenic CD4^+^ T cells for 6 days, **(A)** On day 6, cells were stained with mAb against CD3 and CD4, and T cell proliferation was measured by examining CFSE expression on CD3^+^, CD4^+^ T cells. Data is from one donor and is representative of data from 3 donors. Bar graphs represent the percent of proliferating CD4^+^ CD3^+^ T cells after 6 days in coculture with untreated or treated **(B)** BDCA-1^+^ and **(C)** BDCA-3^+^ mDCs. Data represents the mean + SEM of the percent of proliferating T cells from 3 donors. *= Statistically significant *(p≤0.05)* difference in the percent of proliferating T cells after coculture with infected and uninfected cells as calculated by students paired *t*-test.

BDCA-1^+^ and BDCA-3^+^ mDCs are powerful and equivalent stimulators of allogenic CD4^+^ T cells [13]; however, RSV-infected Mo-DCs demonstrate a decreased capacity to stimulate T-cell proliferation [17,18]. To assess whether RSV infection affects the capacity of BDCA-1^+^ and BDCA-3^+^ mDCs to stimulate T cell proliferation, allogenic CD4^+^ T cells labeled with CFSE were cocultured with BDCA-1 and BDCA-3 mDCs exposed to RSV, or poly I:C for 6 days. T cell proliferation was measured using flow cytometry to examine CFSE expression by CD3^+^, CD4^+^ T cells. As compared to uninfected DCs or those activated with poly I:C, RSV-infected mDCs demonstrate a reduced capacity to stimulate the proliferation of allogenic T cells; UV inactivation of the virus restored the ability of BDCA-1^+^ and BDCA-3^+^ mDCs to stimulate T cell proliferation (Figure 8A-B).

## Discussion

In this study, we examined the effect of RSV infection on primary human mDC subsets isolated from peripheral blood. To ensure purity of the specific mDC populations among donors, BDCA-1^+^ and BDCA-3^+^ mDCs were isolated using multicolor FACs sorting with parameters similar to those previously published [13]. This method allowed us to consistently obtain sufficient numbers of both populations with purity ≥95% for all donors. Similar to Mo-DCs [17,18], we demonstrate that primary mDC subsets are susceptible to infection with RSV. Consistent with previous reports [25], BDCA-1^+^ and BDCA-3^+^ mDCs show similar rates of RSV infection. While mDC activation is not dependent on viral replication, we demonstrate that RSV infection induces a distinct pattern of costimulatory molecule expression and cytokine production by BDCA-1^+^ and BDCA-3^+^ mDCs, and impairs their ability to stimulate T cell proliferation.

Enhanced expression of CD86, CD80, and PD-L1 on RSV-infected mDCs coincides with the costimulatory molecule expression on RSV-infected Mo-DCs and murine lung mDCs which have an impaired capacity to stimulate T cell proliferation [18,19]. These findings suggest that the expression of inhibitory molecules by infected mDCs may contribute to their decreased capacity to stimulate T cell activation. As CD80 is a highly effective ligand for CTLA-4 on activated T cells [37], upregulation of CD80 on BDCA-1^+^ and BDCA-3^+^ mDCs provides a possible mechanism by which RSV could inhibit T cell proliferation. CD86 seems to be more important in CD28 interactions, and in Tregs, CD28 activation provides critical signals needed for proliferation and survival [38,39], thus providing an additional mechanism for the suppression of reactive T cell responses. Our findings of significant levels of CD86 induction by RSV-infected BDCA-3^+^ mDCs may then indicate a differential role for BDCA-1^+^ and BDCA-3^+^ mDCs in Treg homeostasis during RSV infection. However, it is likely the relative expression levels of CD80 and CD86 on mDCs dictates the balance between stimulatory and inhibitory outcomes and work in conjunction to regulate T cell proliferation. The differential expression of PD-L1 by RSV-infected BDCA-1^+^ and BDCA-3^+^ mDCs suggests another mechanism by which RSV infection could impair the ability of mDCs to stimulate T cell proliferation as seen in mice [19].

The cytokine milieu present at the time of TCR activation is crucial in determining the outcome of T cell responses, and DC-derived cytokines and chemokines are at the center of a complex network of cells that regulate these signals. We found that RSV-infected BDCA-1^+^ mDC produced a profile of cytokines and chemokines associated with pro-inflammatory responses, whereas BDCA-3^+^ mDCs did not. Whether the differences in the cytokine profiles produced by RSV-infected BDCA-1^+^ and BDCA-3^+^ mDCs are due to inherent functional differences purported to exist between the subsets, or represents differences in the way RSV interacts with each mDC subtype is unclear. Activation with poly I:C has been shown to induce the production of pro-inflammatory cytokines from both BDCA-1^+^ and BDCA-3^+^ mDCs, as well as the increased production of IL-12(p70) by BDCA-3^+^ mDCs when activated with a cocktail of poly I:C and additional activation signals [13]. However, in contrast to purified toll like receptor agonists, viral pathogens likely activate multiple signaling pathways that lead to alternative patterns of cytokine expression by each cell type. Our findings of IL-12(p70) production by infected BDCA-1^+^ and not BDCA-3^+^ mDCs, may then represent responses specific to infection with RSV. Findings that as compared to activation with poly I;C, RSV infected BDCA-1^+^ mDCs produced greater amounts of proinflammatory and immunoregulatory cytokines, whereas RSV-infected BDCA-3^+^ mDCs produced decreased amounts of proinflammatory cytokines and increased amounts of immunoregulatory cytokines, provides additional evidence for RSV-specific responses. However, the TLRs governing RSV recognition, and their subsequent responses, in primary mDC subsets have yet to be defined. Thus, we cannot exclude the possibility that the differences in cytokine expression between RSV and poly I:C treated DCs are related to differences in the strength or quality of TLR activation by RSV, rather than from a virus-specific effect on cell activation. Further work is needed to determine the cytokine profiles produced by BDCA-1^+^ and BDCA-3^+^ mDCs in response to infection with other viral pathogens.

The balance of pro-inflammatory cytokines, in particular IL-12, produced by RSV-infected BDCA-1^+^ mDCs suggests that BDCA-1^+^ mDCs may promote Th1 responses, whereas RSV-infected BDCA-3^+^ mDCs, which produce IL-10 in the setting of low IL-12, may skew naïve T cells away from Th1 with possible polarization towards Th2 responses. Although mDCs were not found to produce IL-4, RSV-infected BDCA-1^+^ mDCs produced a number of chemokines implicated in the recruitment of cells known to produce IL-4 [40]. The potentially divergent roles of RSV-infected BDCA-1^+^ and BDCA-3^+^ mDCs in the polarization of T cell responses may explain why despite findings of Th2 responses *in vivo*[7,41], *in vitro* studies have not demonstrated the ability of RSV-infected Mo-DCs to bias naïve T cells towards Th2 responses [6,17,42]. Of note, our findings of IL-12 production by BDCA-1^+^ mDCs and the lack of significant production of G-CSF, MCP-1 or MIP-1α by BDCA-3^+^ mDCs are in contrast to a recently published report by Johnson et al. examining the impact of RSV infection on the maturation and cytokine response of primary human mDCs isolated using sequential immunomagnetic selection alone [25]. These differences may be related to their use of a recombinant RSV strain, as well as differences in the methodologies used to isolate and culture mDC subsets. In prior reports, IL-12 and IFN-γ production by Mo-DCs [18] and cord-blood derived DCs [30,31] infected with non-recombinant RSV strains has been demonstrated.

Interestingly, both BDCA-1^+^ and BDCA-3^+^ mDCs produced IL-10 in response to RSV. DC-derived IL-10 is shown to act in an autocrine fashion to inhibit pro-inflammatory cytokine production and impair the capacity of DCs to foster Th1 responses [43]. IL-10 produced by RSV-infected BDCA-1^+^ and BDCA-3^+^ mDCs may then also contribute to the impaired T cell responses seen *in vivo*. In prior reports using RSV-infected Mo-DCs, adding IL-10 blocking antibody did not restore T cell proliferation or function [17]. However, *in vitro* models of E. coli infection demonstrating that infected BDCA-1^+^ mDCs, but not Mo-DCs, impair T cell proliferation in an IL-10 dependent manner [44], provide further evidence that findings in Mo-DCs may not reflect the function of primary mDCs. IL-10 has also been used *in vitro* to generate tolerogenic DCs that are functionally anergic, and capable of generating and expanding Tregs [45,46]. *In vivo* activation of pathways by pathogens that promote IL-10 production by DCs could therefore induce functionally tolerogenic DCs [47]. Tolerogenic DCs are characterized by decreased production of pro-inflammatory cytokines and increased production of IL-10 [46]. Similar findings in RSV-infected BDCA-3^+^ mDCs suggest that they may be functionally tolerogenic. Although RSV-infected BDCA-1^+^ mDCs also produced IL-10, the concomitant induction of IL-12 and other pro-inflammatory cytokines suggests a non-tolerogenic phenotype, which may explain why RSV-infected Mo-DCs were not found to generate T cells with suppressive properties [17]. Studies in murine models of acute RSV infection [48,49] indicate that Tregs play an important role in determining the balance between effective antiviral immunity and controlling harmful immunopathology in the host response against RSV. Our findings suggest a possible mechanism for homeostatic regulation of the Treg compartment by BDCA-3^+^ mDCs during RSV infection.

Similar to studies using Mo-DCs and murine DCs [17-19], RSV infection of BDCA-1^+^ and BDCA-3^+^ mDCs impairs their ability to stimulate CD4^+^ T cell proliferation in a mixed lymphocyte reaction. The lack of inhibition mDCs exposed to UV-inactivated RSV suggests that viral replication is required to trigger these events. The mechanism underlying this inhibitory effect is unknown; however, our findings of differential expression PD-L1 and IL-10 by RSV-infected mDCs suggest that RSV modulates the function of BDCA-1^+^ and BDCA-3^+^ mDCs. Although we did consider that the loss of stimulatory function may be due to reduced viability of infected cells, there was no difference in cell viability between uninfected or RSV-infected mDCs.

To our knowledge, this is one of the first studies to examine the functional responses of highly purified mDC subsets to infection with RSV. We recognize that using a reductionist *in vitro* model does not represent the interactions between the complex network of mDC subsets in the lungs and blood during natural infection and a suitable human model to further study these questions is needed. Furthermore, although RSV primarily affects young children, mDC subsets were isolated from the peripheral blood of healthy adults due to the feasibility of obtaining sufficient volumes of blood from pediatric populations. It is not well known whether age dependent differences in mDC subset function exist [50]. However, despite these limitations, this study provides novel evidence for a virus-specific and subset specific-effect of RSV infection on the functional response of BDCA-1^+^ and BDCA-3^+^ mDCs.

## Conclusions

It is not known what type of mDCs are present in the lungs during natural RSV infection, or how host genetic and environmental factors affect the downstream function of BDCA-1^+^ and BDCA-3^+^ mDCs interacting with virus. However, findings of increased frequencies of BDCA-3^+^ mDCs in the peripheral blood of atopic asthmatics [51] suggest that the balance and function of individual mDC subsets during RSV infection plays a crucial role in determining the short- and long-term sequela of disease. Defining the mechanisms by which RSV is able to modulate virus-specific immune responses will provide important new insights into the immunopathogenesis of RSV infection and the role mDC subsets play in the host immune response against pathogens as a whole.

## Abbreviations

APC: Allophycocyanin; BDCA: Blood Dendritic Cell Antigen; CD: Cluster of differentiation; DCs: Dendritic cells; EDTA: Ethylenediaminetetraacetic acid; FACS: Fluorescence Activated Cell Sorting; FBS: Fetal bovine serum; FITC: Fluorescein isothiocyanate; FSC: Forward scatter; GM-CSF: Granulocyte macrophage colony-stimulating factor; HLA: Human leukocyte antigen; IFN: Interferon; IL: Interleukin; mAb: Monoclonal antibody; MACS: Magnetic Activated Cell Sorting; mDCs: Myeloid dendritic cells; MLR: Mixed lymphocyte reaction; Mo-DCs: Monocyte derived dendritic cells; MOI: Multiplicity of infection; PBMC: Peripheral blood mononuclear cells; pDCs: Plasmacytoid dendritic cells; PE: Phycoerythrin; Per Cp-Cy5.5: Peridinin Chlorophyll Protein Complex conjugated with cyanine dye; RSV: Respiratory syncytial virus; SSC: Side scatter; SEM: Standard error of the mean; Th: T helper cell; TLR: Toll-like Receptor; Treg: T regulatory cells.

## Competing interests

The authors declare that they have no competing interests.

## Authors’ contributions

MRG, DK and RPG designed the research, MRG and DK performed the research, MRG and DK analyzed the data, and MRG, DK, and RPG wrote the manuscript. All authors read and approved the final manuscript.

## Supplementary Material

Additional file 1: Figure S1 Costimulatory expression by mDC subsets exposed to UV-inactivated RSV. BDCA-1^+^ and BDCA-3^+^ mDCs were incubated in media alone, or exposed to RSV and UV-inactivated RSV (non-replicating virus) at a MOI=5 for 40 h. Costimulatory molecule expression was analyzed using flow cytometry. Dashed line= uninfected cells, solid line = RSV-infected cells, dotted line= UV-RSV exposed cells. Click here for file

Additional file 2: Figure S2 Costimulatory molecule expression by mDCs activated with poly I:C. (A) BDCA-1^+^ and (B) BDCA-3^+^ mDCs were isolated and incubated with poly I:C or media for 40 hours. Data represent the mean fluorescent intensity (MFI) of costimulatory molecule expression (mean + SEM of 5 donors) on a linear scale. *=statistically significant difference (*p≤0.05*) in expression by poly I:C-activated mDCs as compared to untreated mDCs by student’s paired *t*-test. Click here for file

Additional file 3: Figure S3 Cytokine production by mDCs activated with poly I:C. (A) BDCA-1^+^ and (B) BDCA-3^+^ mDCs were isolated and incubated with poly I:C or media for 40 hours. Cytokine and chemokine levels were measured in cell-free supernatant by multiplex assay. Data represent cytokine production in pg/ml. Box and whisker plots show the median (central bar), interquartiles (boxes), and range (whiskers) of the data from 5 donors. Linear data is reported on a logarithmic scale. *= Statistically significant *(p≤0.05)* difference in cytokine concentration between treated and untreated cells as calculated by student’s paired *t*-test. Click here for file

## References

[B1] Glezen WP Taber LH Frank AL Kasel JA Risk of primary infection and reinfection with respiratory syncytial virus Am J Dis Child 1986 140 543 546 3706232 10.1001/archpedi.1986.02140200053026

[B2] Dowell SF Anderson LJ Gary HE Jr Erdman DD Plouffe JF File TM Jr Marston BJ Breiman RF Respiratory syncytial virus is an important cause of community-acquired lower respiratory infection among hospitalized adults J Infect Dis 1996 174 456 462 10.1093/infdis/174.3.456 8769600

[B3] Sigurs N Bjarnason R Sigurbergsson F Kjellman B Respiratory syncytial virus bronchiolitis in infancy is an important risk factor for asthma and allergy at age 7 Am J Respir Crit Care Med 2000 161 1501 1507 10.1164/ajrccm.161.5.9906076 10806145

[B4] Tripp R Moore D Barskey A Jones L Moscatiello C Keyserling H Anderson LJ Peripheral blood mononuclear cells from infants hospitalized because of respiratory syncytial virus infection express T helper-1 and T helper-2 cytokines and CC chemokine messenger RNA J Infect Dis 2002 185 1388 1394 10.1086/340505 11992272

[B5] Welliver TP Garofalo RP Hosakote Y Hintz KH Avendano L Sanchez K Velozo L Jafri H Chavez-Bueno S Ogra PL Severe human lower respiratory tract illness caused by respiratory syncytial virus and influenza virus is characterized by the absence of pulmonary cytotoxic lymphocyte responses J Infect Dis 2007 195 1126 1136 10.1086/512615 17357048 PMC7109876

[B6] Munir S Hillyer P Le Nouen C Buchholz UJ Rabin RL Collins PL Bukreyev A Respiratory syncytial virus interferon antagonist NS1 protein suppresses and skews the human T lymphocyte response PLoS Pathog 2011 7 e1001336 e1001336 10.1371/journal.ppat.1001336 21533073 PMC3080852

[B7] Sung RY Hui SH Wong CK Lam CW Yin J A comparison of cytokine responses in respiratory syncytial virus and influenza A infections in infants Eur J Pediatr 2001 160 117 122 10.1007/s004310000676 11271383

[B8] McKenna K Beignon A-S Bhardwaj N Plasmacytoid dendritic cells: linking innate and adaptive immunity J Virol 2005 79 17 27 10.1128/JVI.79.1.17-27.2005 15596797 PMC538703

[B9] Vermaelen K Pauwels R Pulmonary dendritic cells Am J Respir Crit Care Med 2005 172 530 551 10.1164/rccm.200410-1384SO 15879415

[B10] Demedts IK Brusselle GG Vermaelen KY Pauwels R Identification and characterization of human pulmonary dendritic cells Am J Respir Cell Mol Biol 2005 32 177 184 10.1165/rcmb.2004-0279OC 15576669

[B11] Demedts IK Bracke KR Maes T Joos GF Brusselle GG Different roles for human lung dendritic cell subsets in pulmonary immune defense mechanisms Am J Respir Cell Mol Biol 2006 35 387 393 10.1165/rcmb.2005-0382OC 16627825

[B12] Bachem A Guttler S Hartung E Ebstein F Schaefer M Tannert A Salama A Movassaghi K Opitz C Mages HW Superior antigen cross-presentation and XCR1 expression define human CD11c+CD141+ cells as homologues of mouse CD8+ dendritic cells J Exp Med 2010 207 1273 1281 10.1084/jem.20100348 20479115 PMC2882837

[B13] Jongbloed SL Kassianos AJ McDonald KJ Clark GJ Ju X Angel CE Chen C-JJ Dunbar PR Wadley RB Jeet V Human CD141+ (BDCA-3)+ dendritic cells (DCs) represent a unique myeloid DC subset that cross-presents necrotic cell antigens J Exp Med 2010 207 1247 1260 10.1084/jem.20092140 20479116 PMC2882828

[B14] Robbins SH Walzer T Dembele D Thibault C Defays A Bessou G Xu H Vivier E Sellars M Pierre P Novel insights into the relationships between dendritic cell subsets in human and mouse revealed by genome-wide expression profiling Genome Biol 2008 9 R17 R17 10.1186/gb-2008-9-1-r17 18218067 PMC2395256

[B15] Haniffa M Shin A Bigley V McGovern N Teo P See P Wasan PS Wang X-N Malinarich F Malleret B Human tissues contain CD141hi cross-presenting dendritic cells with functional homology to mouse CD103+ nonlymphoid dendritic cells Immunity 2012 37 60 73 10.1016/j.immuni.2012.04.012 22795876 PMC3476529

[B16] Gill M Palucka AK Barton T Ghaffar F Jafri H Banchereau J Ramilo O Mobilization of plasmacytoid and myeloid dendritic cells to mucosal sites in children with respiratory syncytial virus and other viral respiratory infections J Infect Dis 2005 191 1105 1115 10.1086/428589 15747246

[B17] De Graaff PM De Jong EC Van Capel TM Van Dijk ME Roholl PJM Boes J Luytjes W Kimpen JLL Van Bleek GM Respiratory syncytial virus infection of monocyte-derived dendritic cells decreases their capacity to activate CD4 T cells J Immunol 2005 175 5904 5911 16237083 10.4049/jimmunol.175.9.5904

[B18] Guerrero-Plata A Casola A Suarez G Yu X Spetch L Peeples ME Garofalo RP Differential response of dendritic cells to human metapneumovirus and respiratory syncytial virus Am J Respir Cell Mol Biol 2006 34 320 329 10.1165/rcmb.2005-0287OC 16284360 PMC2644197

[B19] Guerrero-Plata A Kolli D Hong C Casola A Garofalo RP Subversion of pulmonary dendritic cell function by paramyxovirus infections J Immunol 2009 182 3072 3083 10.4049/jimmunol.0802262 19234204 PMC2865244

[B20] Sallusto F Lanzavecchia A Efficient presentation of soluble antigen by cultured human dendritic cells is maintained by granulocyte/macrophage colony-stimulating factor plus interleukin 4 and downregulated by tumor necrosis factor alpha J Exp Med 1994 179 1109 1118 10.1084/jem.179.4.1109 8145033 PMC2191432

[B21] Vuckovic S Fearnley DB Mannering SI Dekker J Whyte LF Hart DN Generation of CMRF-44+ monocyte-derived dendritic cells: insights into phenotype and function Exp Hematol 1998 26 1255 1264 9845382

[B22] Thurner B Roder C Dieckmann D Heuer M Kruse M Glaser A Keikavoussi P Kampgen E Bender A Schuler G Generation of large numbers of fully mature and stable dendritic cells from leukapheresis products for clinical application J Immunol Methods 1999 223 1 15 10.1016/S0022-1759(98)00208-7 10037230

[B23] Osugi Y Vuckovic S Hart DN Myeloid blood CD11c(+) dendritic cells and monocyte-derived dendritic cells differ in their ability to stimulate T lymphocytes Blood 2002 100 2858 2866 10.1182/blood.V100.8.2858 12351396

[B24] MacDonald KPA Munster DJ Clark GJ Dzionek A Schmitz J Hart DNJ Characterization of human blood dendritic cell subsets Blood 2002 100 4512 4520 10.1182/blood-2001-11-0097 12393628

[B25] Johnson TR Johnson CN Corbett KS Edwards GC Graham BS Primary human mDC1, mDC2, and pDC dendritic cells are differentially infected and activated by respiratory syncytial virus PLoS One 2011 6 e16458 e16458 10.1371/journal.pone.0016458 21297989 PMC3030580

[B26] Probst HC McCoy K Okazaki T Honjo T van den Broek M Resting dendritic cells induce peripheral CD8+ T cell tolerance through PD-1 and CTLA-4 Nat Immunol 2005 6 280 286 15685176 10.1038/ni1165

[B27] Beswick EJ Pinchuk IV Das S Powell DW Reyes VE Expression of the programmed death ligand 1, B7-H1, on gastric epithelial cells after Helicobacter pylori exposure promotes development of CD4+ CD25+ FoxP3+ regulatory T cells Infect Immun 2007 75 4334 4341 10.1128/IAI.00553-07 17562772 PMC1951191

[B28] Krupnick AS Gelman AE Barchet W Richardson S Kreisel FH Turka LA Colonna M Patterson GA Kreisel D Murine vascular endothelium activates and induces the generation of allogeneic CD4+25+Foxp3+ regulatory T cells J Immunol 2005 175 6265 6270 16272276 10.4049/jimmunol.175.10.6265

[B29] Pulendran B Variegation of the immune response with dendritic cells and pathogen recognition receptors J Immunol 2005 174 2457 2465 15728447 10.4049/jimmunol.174.5.2457

[B30] Bartz H Buning-Pfaue F Turkel O Schauer U Respiratory syncytial virus induces prostaglandin E2, IL-10 and IL-11 generation in antigen presenting cells Clin Exp Immunol 2002 129 438 445 10.1046/j.1365-2249.2002.01927.x 12197884 PMC1906469

[B31] Chi B Dickensheets HL Spann KM Alston MA Luongo C Dumoutier L Huang J Renauld JC Kotenko SV Roederer M Alpha and lambda interferon together mediate suppression of CD4 T cells induced by respiratory syncytial virus J Virol 2006 80 5032 5040 10.1128/JVI.80.10.5032-5040.2006 16641294 PMC1472058

[B32] De Jong EC Smits HH Kapsenberg ML Dendritic cell-mediated T cell polarization Springer Semin Immunopathol 2005 26 289 307 10.1007/s00281-004-0167-1 15609003

[B33] Benwell RK Hruska JE Fritsche KL Lee DR Double stranded RNA- relative to other TLR ligand-activated dendritic cells induce extremely polarized human Th1 responses Cell Immunol 2010 264 119 126 10.1016/j.cellimm.2010.05.008 20547386

[B34] Sa L Cyster JG Chemokines as regulators of T cell differentiation Nat Immunol 2001 2 102 107 10.1038/84205 11175801

[B35] Sporri R Reis e Sousa C Inflammatory mediators are insufficient for full dendritic cell activation and promote expansion of CD4+ T cell populations lacking helper function Nat Immunol 2005 6 163 170 15654341 10.1038/ni1162

[B36] Rajkovic I Dragicevic A Vasilijic S Bozic B Dzopalic T Tomic S Majstorovic I Vucevic D Djokic J Balint B Colic M Differences in T-helper polarizing capability between human monocyte-derived dendritic cells and monocyte-derived Langerhans'-like cells Immunology 2011 132 217 225 10.1111/j.1365-2567.2010.03356.x 21039466 PMC3050445

[B37] Collins AV Brodie DW Gilbert RJ Iaboni A Manso-Sancho R Walse B Stuart DI van der Merwe PA Davis SJ The interaction properties of costimulatory molecules revisited Immunity 2002 17 201 210 10.1016/S1074-7613(02)00362-X 12196291

[B38] Zheng Y Manzotti CN Liu M Burke F Mead KI Sansom DM CD86 and CD80 differentially modulate the suppressive function of human regulatory T cells J Immunol 2004 172 2778 2784 14978077 10.4049/jimmunol.172.5.2778

[B39] Tang Q Henriksen KJ Boden EK Tooley AJ Ye J Subudhi SK Zheng XX Strom TB Bluestone JA Cutting edge: CD28 controls peripheral homeostasis of CD4+CD25+ regulatory T cells J Immunol 2003 171 3348 3352 14500627 10.4049/jimmunol.171.7.3348

[B40] Commins SP Borish L Steinke JW Immunologic messenger molecules: cytokines, interferons, and chemokines J Allergy Clin Immunol 2010 125 S53 S72 10.1016/j.jaci.2009.07.008 19932918

[B41] Garofalo RP Patti J Hintz K Hill V Ogra PL Welliver RC Macrophage inflammatory protein-1alpha (not T helper type 2 cytokines) is associated with severe forms of respiratory syncytial virus bronchiolitis J Infect Dis 2001 184 393 399 10.1086/322788 11471095

[B42] Le Nouen C Hillyer P Munir S Winter CC McCarty T Bukreyev A Collins PL Rabin RL Buchholz UJ Effects of human respiratory syncytial virus, metapneumovirus, parainfluenza virus 3 and influenza virus on CD4+ T cell activation by dendritic cells PLoS One 2010 5 e15017 e15017 10.1371/journal.pone.0015017 21124776 PMC2993941

[B43] Corinti S Albanesi C La Sala A Pastore S Girolomoni G Regulatory activity of autocrine IL-10 on dendritic cell functions J Immunol 2001 166 4312 4318 11254683 10.4049/jimmunol.166.7.4312

[B44] Kassianos AJ Hardy MY Ju X Vijayan D Ding Y Vulink AJ McDonald KJ Jongbloed SL Wadley RB Wells C Human CD1c (BDCA-1)+ myeloid dendritic cells secrete IL-10 and display an immuno-regulatory phenotype and function in response to Escherichia coli Eur J Immunol 2012 42 1512 1522 10.1002/eji.201142098 22678905

[B45] Gregori S Tomasoni D Pacciani V Scirpoli M Battaglia M Magnani CF Hauben E Roncarolo MG Differentiation of type 1 T regulatory cells (Tr1) by tolerogenic DC-10 requires the IL-10-dependent ILT4/HLA-G pathway Blood 2010 116 935 944 10.1182/blood-2009-07-234872 20448110

[B46] Boks MA Kager-Groenland JR Haasjes MS Zwaginga JJ Van Ham SM Ten Brinke A IL-10-generated tolerogenic dendritic cells are optimal for functional regulatory T cell induction–a comparative study of human clinical-applicable DC Clin Immunol 2012 142 332 342 10.1016/j.clim.2011.11.011 22225835

[B47] McGuirk P McCann C Mills KH Pathogen-specific T regulatory 1 cells induced in the respiratory tract by a bacterial molecule that stimulates interleukin 10 production by dendritic cells: a novel strategy for evasion of protective T helper type 1 responses by Bordetella pertussis J Exp Med 2002 195 221 231 10.1084/jem.20011288 11805149 PMC2193600

[B48] Fulton RB Meyerholz DK Varga SM Foxp3+ CD4 regulatory T cells limit pulmonary immunopathology by modulating the CD8 T cell response during respiratory syncytial virus infection J Immunol 2010 185 2382 2392 10.4049/jimmunol.1000423 20639494 PMC2923480

[B49] Loebbermann J Thornton H Durant L Sparwasser T Webster KE Sprent J Culley FJ Johansson C Openshaw PJ Regulatory T cells expressing granzyme B play a critical role in controlling lung inflammation during acute viral infection Mucosal Immunol 2012 5 161 172 10.1038/mi.2011.62 22236998 PMC3282434

[B50] Jyonouchi H Cui C Geng L Yin Z Fitzgerald-Bocarsly P Age-dependent changes in peripheral blood dendritic cell subsets in normal children and children with specific polysaccharide antibody deficiency (SPAD) Eur J Pediatr 2010 169 1233 1239 10.1007/s00431-010-1210-y 20473522 PMC3579493

[B51] Yerkovich ST Roponen M Smith ME McKenna K Bosco A Subrata LS Mamessier E Wikstrom ME Le Souef P Sly PD Allergen-enhanced thrombomodulin (blood dendritic cell antigen 3, CD141) expression on dendritic cells is associated with a TH2-skewed immune response J Allergy Clin Immunol 2009 123 209 216 e204 10.1016/j.jaci.2008.09.009 18947863

