# Supplementary figures and images for: Differential response of BDCA-1+ and BDCA-3+ myeloid dendritic cells to respiratory syncytial virus infection
Source: Respir Res. 2013 Jul 5;14(1):71. doi: 10.1186/1465-9921-14-71 (PMC3708742; doi:10.1186/1465-9921-14-71)

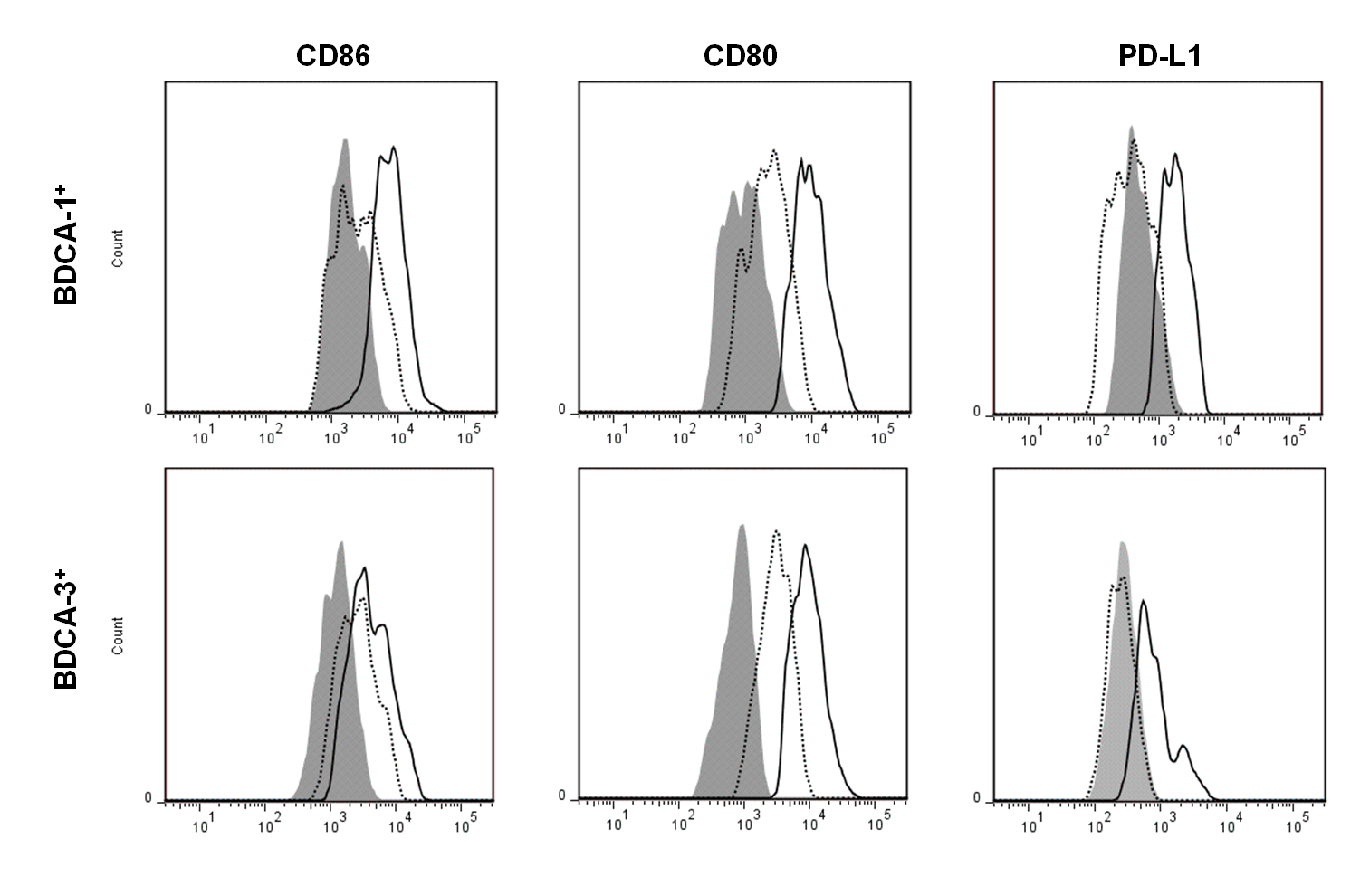

Supplement: Additional file 1: Figure S1 — Costimulatory expression by mDC subsets exposed to UV-inactivated RSV. BDCA-1+ and BDCA-3+ mDCs were incubated in media alone, or exposed to RSV and UV-inactivated RSV (non-replicating virus) at a MOI=5 for 40 h. Costimulatory molecule expression was analyzed using flow cytometry. Dashed line= uninfected cells, solid line = RSV-infected cells, dotted line= UV-RSV exposed cells. [file 1465-9921-14-71-S1.tiff]

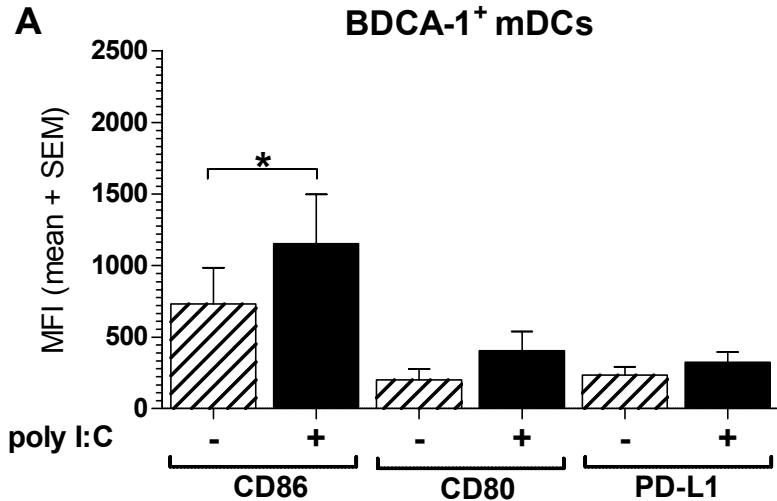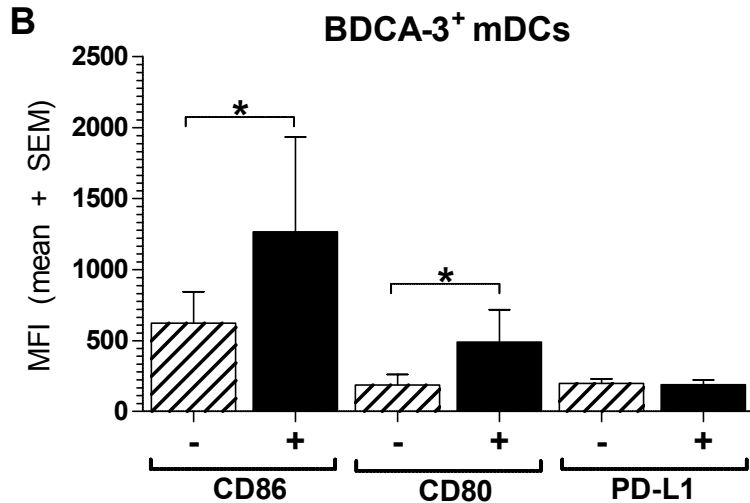

Supplement: Additional file 2: Figure S2 — Costimulatory molecule expression by mDCs activated with poly I:C. (A) BDCA-1+ and (B) BDCA-3+ mDCs were isolated and incubated with poly I:C or media for 40 hours. Data represent the mean fluorescent intensity (MFI) of costimulatory molecule expression (mean + SEM of 5 donors) on a linear scale. *=statistically significant difference (p≤0.05) in expression by poly I:C-activated mDCs as compared to untreated mDCs by student’s paired t-test. [file 1465-9921-14-71-S2.pdf]

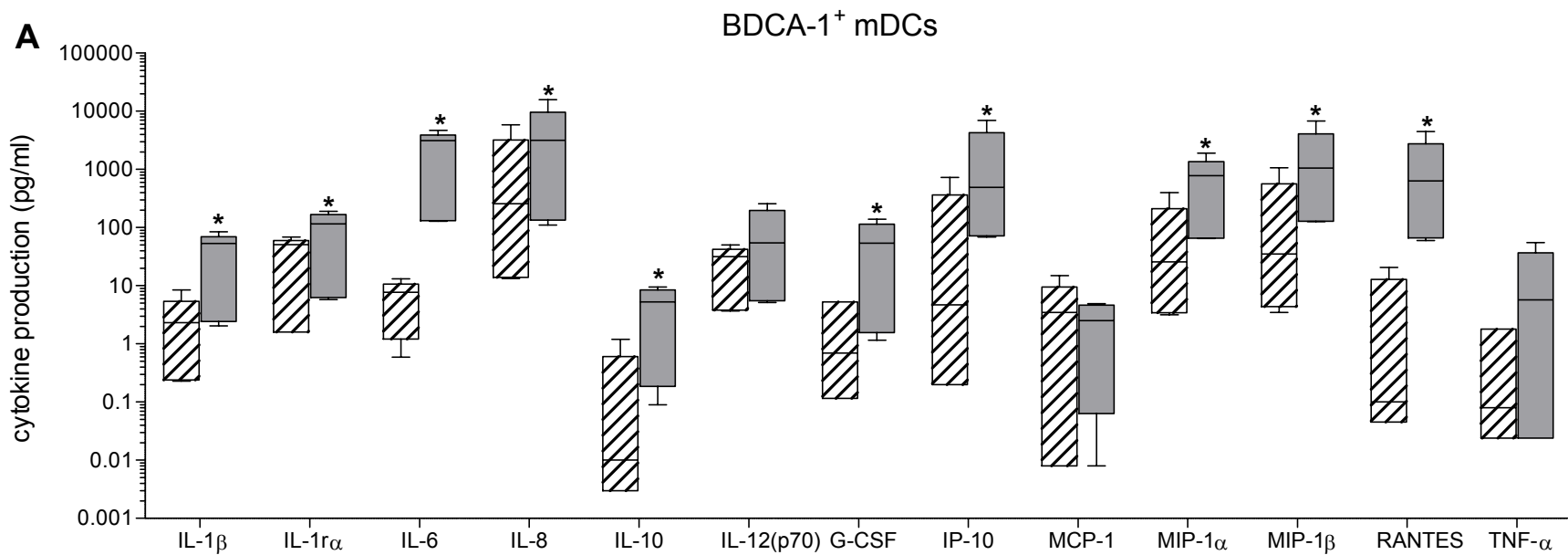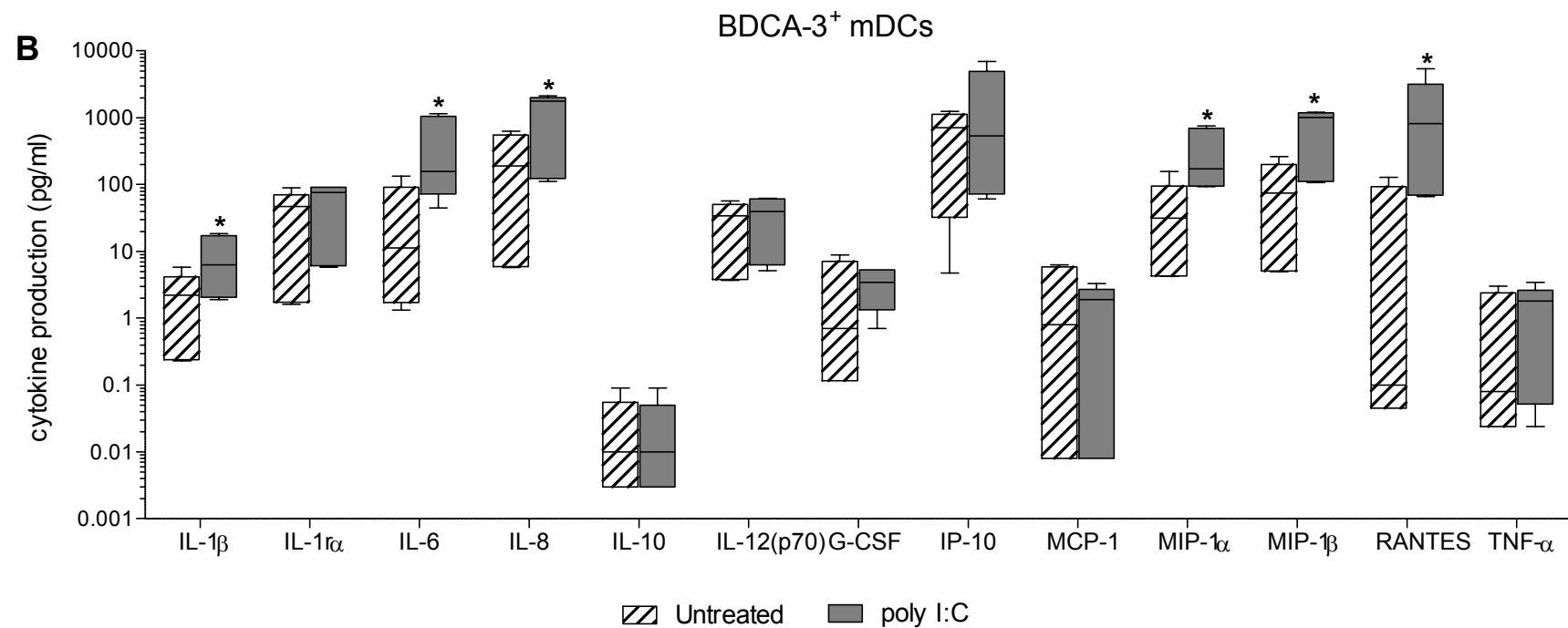

Supplement: Additional file 3: Figure S3 — Cytokine production by mDCs activated with poly I:C. (A) BDCA-1+ and (B) BDCA-3+ mDCs were isolated and incubated with poly I:C or media for 40 hours. Cytokine and chemokine levels were measured in cell-free supernatant by multiplex assay. Data represent cytokine production in pg/ml. Box and whisker plots show the median (central bar), interquartiles (boxes), and range (whiskers) of the data from 5 donors. Linear data is reported on a logarithmic scale. *= Statistically significant (p≤0.05) difference in cytokine concentration between treated and untreated cells as calculated by student’s paired t-test. [file 1465-9921-14-71-S3.pdf]
